# A Value-Based Comparison of the Management of Ambulatory Respiratory Diseases in Walk-in Clinics, Primary Care Practices, and Emergency Departments: Protocol for a Multicenter Prospective Cohort Study

**DOI:** 10.2196/25619

**Published:** 2021-02-22

**Authors:** Simon Berthelot, Mylaine Breton, Jason Robert Guertin, Patrick Michel Archambault, Elyse Berger Pelletier, Danielle Blouin, Bjug Borgundvaag, Arnaud Duhoux, Laurie Harvey Labbé, Maude Laberge, Philippe Lachapelle, Lauren Lapointe-Shaw, Géraldine Layani, Gabrielle Lefebvre, Myriam Mallet, Deborah Matthews, Kerry McBrien, Shelley McLeod, Eric Mercier, Alexandre Messier, Lynne Moore, Judy Morris, Kathleen Morris, Howard Ovens, Paul Pageau, Jean-Sébastien Paquette, Jeffrey Perry, Michael Schull, Mathieu Simon, David Simonyan, Henry Thomas Stelfox, Denis Talbot, Samuel Vaillancourt

**Affiliations:** 1 Axe Santé des populations et Pratiques optimales en santé Centre de recherche du CHU de Québec-Université Laval Québec, QC Canada; 2 Department of Family and Emergency Medicine Université Laval Québec, QC Canada; 3 Department of Community Health sciences Université de Sherbrooke Campus de Longueuil Longueuil, QC Canada; 4 Centre de recherche Charles-Le Moyne - Saguenay-Lac-Saint-Jean sur les innovations en santé Longueuil, QC Canada; 5 Department of Social and Preventive Medicine Université Laval Québec, QC Canada; 6 VITAM - Centre de recherche en santé durable Québec, QC Canada; 7 Centre de recherche du Centre intégré de santé et de services sociaux de Chaudière-Appalaches Lévis, QC Canada; 8 Ministère de la santé et des services sociaux Gouvernement du Québec Québec, QC Canada; 9 Department of Emergency Medicine Queen's University Kingston, ON Canada; 10 Department of Family and Community Medicine University of Toronto Toronto, ON Canada; 11 Schwartz/Reisman Emergency Medicine Institute Sinai Health System Toronto, ON Canada; 12 Faculty of Nursing Université de Montréal Montréal, QC Canada; 13 Operations and Decision Systems Department Faculty of Administrative Sciences Université Laval Québec, QC Canada; 14 Department of Medicine University of Toronto Toronto, ON Canada; 15 Department of Family and Emergency Medicine Université de Montréal Montréal, QC Canada; 16 Ministry of Health and Long Term Care Government of Ontario Toronto, ON Canada; 17 Departments of Family Medicine and Community Health Sciences University of Calgary Calgary, AB Canada; 18 Division of Emergency Medicine Department of Family and Community Medicine University of Toronto Toronto, ON Canada; 19 Hôpital du Sacré-Coeur-de-Montréal Centre intégré universitaire de santé et de services sociaux du Nord-de-l'Île-de Montréal Montréal, QC Canada; 20 Canadian Institute for Health Information Ottawa, ON Canada; 21 Department of Emergency Medicine University of Ottawa Ottawa, ON Canada; 22 Laboratoire ARIMED GMF-U de Saint-Charles-Borromée Québec, QC Canada; 23 Clinical Epidemiology Program Ottawa Hospital Research Institute Ottawa, ON Canada; 24 Department of Emergency Medicine Sunnybrook Research Institute University of Toronto Toronto, ON Canada; 25 Institut universitaire de cardiologie et de pneumologie de Québec Québec, QC Canada; 26 Department of Critical Care Medicine, Medicine and Community Health Sciences, O'Brien Institute for Public Health University of Calgary Calgary, AB Canada; 27 Department of Emergency Medicine St Michael's Hospital Unity Health Toronto Toronto, ON Canada

**Keywords:** emergency department, primary care, walk-in clinic, health economics, quality of care, patient preferences, patient-reported outcomes, outcome assessment, health care

## Abstract

**Background:**

In Canada, 30%-60% of patients presenting to emergency departments are ambulatory. This category has been labeled as a source of emergency department overuse. Acting on the presumption that primary care practices and walk-in clinics offer equivalent care at a lower cost, governments have invested massively in improving access to these alternative settings in the hope that patients would present there instead when possible, thereby reducing the load on emergency departments. Data in support of this approach remain scarce and equivocal.

**Objective:**

The aim of this study is to compare the value of care received in emergency departments, walk-in clinics, and primary care practices by ambulatory patients with upper respiratory tract infection, sinusitis, otitis media, tonsillitis, pharyngitis, bronchitis, influenza-like illness, pneumonia, acute asthma, or acute exacerbation of chronic obstructive pulmonary disease.

**Methods:**

A multicenter prospective cohort study will be performed in Ontario and Québec. In phase 1, a time-driven activity-based costing method will be applied at each of the 15 study sites. This method uses time as a cost driver to allocate direct costs (eg, medication), consumable expenditures (eg, needles), overhead costs (eg, building maintenance), and physician charges to patient care. Thus, the cost of a care episode will be proportional to the time spent receiving the care. At the end of this phase, a list of care process costs will be generated and used to calculate the cost of each consultation during phase 2, in which a prospective cohort of patients will be monitored to compare the care received in each setting. Patients aged 18 years and older, ambulatory throughout the care episode, and discharged to home with one of the aforementioned targeted diagnoses will be considered. The estimated sample size is 1485 patients. The 3 types of care settings will be compared on the basis of primary outcomes in terms of the proportion of return visits to any site 3 and 7 days after the initial visit and the mean cost of care. The secondary outcomes measured will include scores on patient-reported outcome and experience measures and mean costs borne wholly by patients. We will use multilevel generalized linear models to compare the care settings and an overlap weights approach to adjust for confounding factors related to age, sex, gender, ethnicity, comorbidities, registration with a family physician, socioeconomic status, and severity of illness.

**Results:**

Phase 1 will begin in 2021 and phase 2, in 2023. The results will be available in 2025.

**Conclusions:**

The end point of our program will be for deciders, patients, and care providers to be able to determine the most appropriate care setting for the management of ambulatory emergency respiratory conditions, based on the quality and cost of care associated with each alternative.

**International Registered Report Identifier (IRRID):**

PRR1-10.2196/25619

## Introduction

### The Problem: Emergency Department Overuse and Misuse

Emergency departments (EDs) are specialized and costly resources designed to provide care for patients with urgent or life-threatening conditions [1]. In Canada, low-acuity ambulatory patients, who do not require a gurney or constant observation, represent 30%-60% of all ED visits [2-7]. This situation is increasingly considered as overuse and misuse of ED resources and a threat to the quality of care received by patients whose needs are more urgent [8]. Delays experienced in an overcrowded ED can lead to mortality, morbidity, and reduced quality of life [9-14]. ED overcrowding is widely regarded as a serious but largely avoidable public health risk exacerbated by ambulatory patients [15,16].

### An Important Policy Issue

Many Canadian regional health authorities have developed policies so that low-acuity ambulatory emergency patients preferably present to walk-in clinics or primary care practices [17-19]. Over the past decade, numerous innovations have been implemented to improve timely access to primary care, such as extended walk-in clinic hours [17-19] and the advanced access model (timely access to a care provider) for registered patients [20-26]. In Ontario and Québec, governments have invested massively in new models of primary care to improve access to emergency care and thereby decrease ED visits by patients who are treatable in non-ED settings [18,27]. These health policy priorities rely on the assumption that walk-in clinics and primary care facilities offer less costly, more accessible, and more efficient alternatives to the local population [17,28,29] than overcrowded EDs [3,4,7,8,30-34]. As reasonable as this assumption may appear, data supporting it are scarce and equivocal [35,36].

### Determining the Best Care Setting for Ambulatory Emergency Patients: A Knowledge Gap

Few studies have tested the hypothesis that walk-in or primary care clinics offer better care than EDs to ambulatory patients with acute health concerns.

#### The Costs

A prospective study in Ontario in 2005 [28] concluded that for similar cases, ED costs were 3 to 4 times higher than the costs incurred in a family physician’s office or a walk-in clinic. However, compared costs were not adjusted for comorbidities or severity of disease and did not include out-of-pocket expenses (eg, parking) and indirect costs to patients (eg, loss of income). Other studies, mainly from the United States, have reached similar conclusions [17,37-39] but using charges as proxies of health care costs, which has been shown to be an inaccurate costing method [40,41]. Some reports even suggest that walk-in clinics may in fact increase overall health care costs by duplicating care with frequent return visits after an initial visit [42-45].

#### The Quality of Care

Very few studies have considered quality of care and patient health outcomes in determining the best alternative setting for treating ambulatory emergency patients [8]. A 2017 review (Cochrane) of prospective studies comparing mortality, morbidity, and adherence to practice guidelines in walk-in clinics, primary care practices, and EDs found that none met this criterion [46]. However, three retrospective studies [47-49] and one study evaluating costs and return visits [28] suggested that (1) inappropriate use of antibiotics for self-resolving acute respiratory conditions occurs more frequently following visits to urgent care centers and family medicine offices than to EDs [47-50]; (2) the choice of antibiotics is more concordant with practice guidelines in walk-in clinics than in EDs and family medicine practices [48]; and (3) return visit likelihood within 72 hours is higher after an ED care episode than after any other outpatient clinic visit [28]. However, these fragmented and incomplete data come mostly from the United States. A comprehensive research program comparing acute care received in EDs, walk-in clinics, and primary care practices in Canada is long overdue.

#### The Patient Perspective

Deciders often prioritize certain care settings based on potential cost savings, *auctioning off* care paths to the lowest bidder from the government’s perspective [51]. However, studies have shown that from a patient’s perspective, the choice to seek care in either a primary care practice, a walk-in clinic, or an ED is determined not only by ease of primary care access but also by factors such as convenience and perceived severity of illness and previous health care experiences [35,52-56]. What patients value the most differs considerably from what other stakeholders tend to value [57]. The patient’s perspective must be considered to determine the best ambulatory emergency care option. To our knowledge, no studies have compared these alternative settings from a patient’s perspective.

### Conceptual Framework: A Value-Based Approach

To compare the different care setting possibilities for ambulatory emergency patients, we propose value-based assessment, an approach first described by Michael Porter in 2006 [58,59] and widely adopted since by researchers and health quality organizations around the world [60-66]. Value is defined in terms of health outcomes achieved per dollar spent [58,67,68]. It promotes the best care at the lowest cost, without isolating clinical issues from economic issues. Two essential components are needed: (1) a feasible and reliable costing method and (2) valid, reliable, and readily available outcome indicators, consistent with the priorities of patients, deciders, and care providers. This comprehensive paradigm aligns patients, deciders, and clinicians behind shared goals, based on patient preferences and scientific evidence.

### Previous Preliminary Work

Our team has conducted a pilot study in which an ED and a primary care clinic offering walk-in services for frequent ambulatory acute conditions were compared in terms of costs of care and compliance with practice guidelines [69,70]. We reviewed the medical records of 918 adults with one of 13 targeted ambulatory acute conditions during the 2015 and 2016 fiscal year and applied a time-driven activity-based costing method. Time-driven activity-based costing has been found to provide more precise accounting than methods based on diagnosis-related groups and is simpler than conventional activity-based costing [41,71-73]. It assumes that the cost of a care episode is proportional to the time that the patient spends receiving the care. Costs of care are determined by allocating all direct costs (eg, staff salaries) and overhead (eg, building maintenance) to activities related to patient care, including physician charges [74,75]. This costing method has been used successfully in many care settings [65,71,76,77], and we adapted it for use in EDs and primary care practices [41,69,78,79]. The adjusted mean costs in each clinical setting for upper respiratory tract infection (URTI), a condition for which antibiotics and x-rays are generally not recommended [80,81] were determined and the clinical settings were compared on the basis of the process of care applied (Table 1).

**Table 1:** Mean cost of care and percentage of use of nonrecommended care applied to upper respiratory tract infection in a primary care practice and an emergency department.

Variable		Primary care practice (n=102)	Emergency department (n=52)	*P* value
Cost of care^a^ (US $), (mean 95% CI)		45.4 (38.4-53.4)	59.8 (49.4-72.3)	<.001
**Process of care, % (95% CI)**				
	Chest x-ray	13.7 (7.7-22.0)	26.9 (15.6-41.0)	.05
	Antibiotics	44.1 (34.3-54.3)	5.8 (1.2-16.0)	<.001

^a^Mean value adjusted for age, sex, vital signs, comorbidities, and number of regular medications for upper respiratory tract infection.

On the basis of this preliminary study, we conclude that (1) time-driven activity-based costing is feasible in ED and primary care settings without requiring advanced information technologies or rigorously coded electronic medical records, 2 major barriers to conducting research in outpatient clinics, and (2) significant variations in costs and quality of care may exist between EDs and clinics, suggesting that a multicenter cohort study is warranted. However, this retrospective study highlighted major issues that only a prospective design can resolve: comorbidities (crucial to risk adjustment), disposition plans (crucial to assessing quality of care), and discharge diagnosis are not readily extractable from databases in the outpatient setting and are often missing or incomplete in medical notes. By manually reviewing thousands of visits logged in electronic records, our research assistants identified eligible cases one chart at a time. These major hurdles apply to outpatient clinics in all Canadian provinces. A retrospective design for a multicenter cross-jurisdictional study would have major methodological flaws because of the unlikeliness of obtaining comparable information across settings. More importantly, a retrospective study on administrative databases would not allow us to assess patients’ perspectives. Finally, a randomized controlled trial is not feasible for the population and settings under study because randomization would have to occur before any contact with the health system to assign patients to their treatment group. For these reasons, we believe that a prospective cohort study is the most appropriate design for identifying the best care setting for ambulatory emergency patients.

### Objectives

Our goal is to compare the health outcomes and costs of care received in EDs, walk-in clinics, and primary care practices by ambulatory patients presenting with acute respiratory conditions, namely, URTI, sinusitis, otitis media, pharyngitis, tonsillitis, bronchitis, influenza-like illness, pneumonia, acute asthma, or acute exacerbation of chronic obstructive pulmonary disease (COPD). We selected these conditions because many performance metrics have been validated previously for assessing the quality of care provided [82,83]. Highly prevalent in ambulatory emergency care before the COVID-19 pandemic [16,50], acute respiratory conditions are now putting even greater strain on already overstretched health care systems. In addition, the pandemic has shifted primary care services significantly toward telemedicine (ie, remote consultation by phone or videoconferencing) [84]. Determining where these patients can get the most effective care is a crucial issue. Our 3 specific objectives are to (1) estimate the costs of care processes administered by care providers in EDs, walk-in clinics, and primary care practices for acute respiratory conditions from the public payer’s perspective; (2) estimate and compare the cost of care episodes in EDs, walk-in clinics, and primary care practices for acute respiratory conditions from the public payer’s and patient’s perspectives; and (3) compare patient health outcomes and quality of care in these care settings when treating acute ambulatory respiratory conditions from the public payer’s and patient’s perspectives. To achieve these objectives, we propose a 4-year (from April 1, 2021, to March 31, 2025) research plan in 2 phases: a time-driven activity-based costing method study (objective 1) and a prospective cohort study (objectives 2 and 3).

## Methods

### Phase 1: A Time-Driven Activity-Based Costing Method Study

#### Setting

A time-driven activity-based costing study will first be performed for fiscal year April 1, 2021, to March 31, 2022. We shall estimate the cost of care processes administered by care providers (Objective 1) in 3 different models of ambulatory emergency care in Québec and Ontario: (1) discontinuous care in the ED (by physicians unfamiliar with the patients); (2) discontinuous care in a walk-in clinic (by physicians unfamiliar with the patients); and (3) continuous care in primary care clinic (patients attached to a primary care practice, seen by their family physician or a colleague on a same-day appointment for urgent needs).

We have confirmed the participation of 14 of the 15 planned patient recruitment sites (ED 5/5, walk-in 5/5, primary care 4/5; Multimedia Appendix 1). They have been selected in different types of urban areas, including small (Joliette), medium (Kingston), large (Québec City, Ottawa), and metropolitan (Montreal) cities. In each participating region, an ED will be paired with a nearby walk-in clinic and primary care practice. We are currently securing our final additional clinic in Ottawa with the help of BeACCoN (Better Access and Care for Complex Needs), a provincial primary care research network.

#### Design

**Figure 1:**
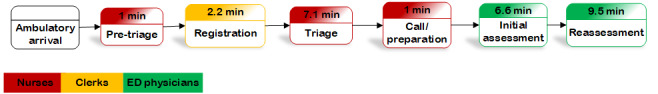
Process mapping for upper respiratory tract infections in the emergency department (truncated). Each box represents a process with its duration. Colors identify human resources (red=nurses; yellow=clerks; green=physicians). ED: emergency department.

The time-driven activity-based costing method [72,85] will enable us to derive for each setting the cost of care processes (eg, triage) and traceable supplies (eg, medication) potentially provided to patients with acute respiratory conditions, which includes telemedicine. This costing method requires only 2 parameters, namely, the unit cost of supplying capacity and the duration of processes, and comprises the following steps:

1. Process (eg, salbutamol in acute asthma) and resource (eg, respiratory therapist) mapping through discussion with local teams for each respiratory condition (Figure 1)
2. Validation of process maps and durations by on-field research assistants prospectively observing patients and measuring process duration using time-motion software (UMT Plus [Laubrass])
3. Calculation, with local administrative teams, of total annual overhead costs (eg, building maintenance) related to the care of ambulatory patients with acute conditions (Multimedia Appendix 2 for allocation rules)
4. Estimation of cost per time unit ($/minute) for the following cost elements obtained by dividing yearly expenses for a cost element by the total yearly number of minutes worked by professionals to care for patients in this facility (Multimedia Appendix 3): (1) human resources (eg, nurses, physicians) or equipment (eg, x-ray machine), (2) consumable supplies (eg, gloves, needles, paper), and (3) overhead costs
5. Estimation of the cost of traceable supplies (eg, laboratory testing)
6. Calculation of the average cost of each health care process (Multimedia Appendicies 3 and 4)

The cost of a care process is proportional to the mean duration measured on field. For example, the cost of triage is estimated by adding up the expenses associated with the triage nurse, consumables, and overhead. These elements will be estimated by multiplying the mean triage duration by their unit costs as follows:

Cost of triage = mean triage duration × (unit cost of nurse + unit cost of consumables+ unit cost of overhead) = 7.1 min × (US $0.78/min + US $0.07/min + US $0.17/min) = US $7.24 (Can $1 [US $0.76])

The cost of telemedicine will be estimated following the same steps, from resource mapping and time measurement through allocation of overhead and consumables, all the way to average cost calculation.

Where applicable, the following adjustments will be made so that the estimated costs reflect the public payer’s perspective: (1) expenses paid by physicians or owners of a participating clinic will be subtracted from the yearly expenses related to the appropriate cost element (eg, salaries, overhead); (2) similarly, government funding received by a clinic apart from physician remuneration will be added.

#### Financial Data Sources

The accounting department at each participating site will provide all financial data, except for physician charges. To calculate the unit cost for physicians, the total amount charged by all physicians per site per year will be obtained from local private billing agencies. This sum will be divided by the number of minutes spent delivering patient care, which will be obtained from physician schedules.

#### Intermediate Outputs of Phase 1

In addition to institution-specific costs, upon completing phase 1, we will create a list of standardized costs of care for each process and associated traceable supplies based on the average costs estimated in the 15 institutions (ED, walk-in, primary care, both provinces). Use of standardized costs will eliminate price effects because of differential costings between sites and provinces, thereby facilitating comparisons between the 3 clinical settings. In phase 2, the cost of a care episode will be calculated per individual by summing the standardized costs of care processes, supplies, and drugs received by each patient during their visit. Fixed and variable costs will be broken down to estimate and compare the care settings in terms of the marginal cost of each new patient assessed [86].

### Phase 2: A Prospective Cohort for Comparing the 3 Health Care Settings

#### Design and Setting

A multicenter prospective cohort study will be conducted in the institutions included in phase 1 to compare the value of care in EDs, walk-in clinics, and primary care practices (Objectives 2 and 3).

#### Selection of Participants

We shall include patients (1) aged 18 years and older; (2) seen in person or via telemedicine in an ED, a walk-in clinic, or the primary care practice where they are registered; (3) ambulatory during the entire visit or consultation; and (4) discharged home with a diagnosis of URTI, sinusitis, otitis media, pharyngitis, tonsillitis, bronchitis, influenza-like illness, pneumonia, acute asthma, or acute exacerbation of COPD. We shall exclude patients (1) transported by ambulance, (2) not covered by the provincial health insurance plan, (3) having consulted for a similar problem in the previous 30 days as patients with refractory diseases representing a population with different care needs, (4) living in a long-term health care facility or incarcerated, or (5) receiving palliative care.

#### Recruitment Procedures on the Initial Visit

A research nurse in collaboration with local clerks at each site will screen eligible patients after on-site registration or web-based scheduling, but before assessment by a physician, based on presenting complaints suggestive of acute respiratory conditions. After assessment and once a targeted diagnosis is confirmed, the same research nurse will prospectively (1) obtain consent from patients; (2) ensure that the discharge diagnosis, comorbidities, and disposition plans are fully documented; and (3) administer a questionnaire to assess patient experience of care and motivation for choosing one care setting over the other. Motivation will be classified into the 6 domains of the Conceptual Model of Emergency Department Use [35]. Participants will be asked to specify whether their choice of care setting was based on accessibility, convenience, their perception of the severity of illness, their beliefs and knowledge regarding these care settings, referral and advice from a care professional or an acquaintance, or costs. They will also be requested to rate their perception of illness severity. For on-site participants only, the research nurse will also (1) assess the severity using the Pandemic Medical Early Warning Score (PMEWS), a validated severity score allowing points for age, vital signs, comorbidity, social situation, and functional status [87], and (2) perform spirometry (measured parameter: forced expiratory volume in the first second [FEV_1_]) on patients with acute asthma. A random sampling recruitment schedule will be planned to ensure a proportional representation of the hours of operation for each recruiting site. Participating EDs will recruit on a schedule similar to their paired participating clinics to include participants who could have consulted in an alternative setting and exclude night patients who differ significantly from patients seeking care during the day [88]. Recruitment will occur over a full year to encompass seasonal variability in the incidence of respiratory diseases.

#### Data Collection and Follow-Up Phone Calls

Research assistants at each site will complete data collection from local medical records. For on-site participants and, where appropriate, for those assessed by telemedicine, they will compile the following information: age, sex, gender, ethnicity, postal code, distance from facility to home, referral by the provincial telephone consultation service (811, Telehealth), enrollment with a family physician, presenting complaints, comorbidities, regular medications, date and time of arrival and discharge, vital signs upon arrival, investigations and interventions during care episode, discharge diagnosis, and prescriptions upon discharge. A follow-up phone call will be made to all participants 10 days after the initial visit to collect data initially unavailable in medical records and to evaluate primary and secondary outcome metrics. Patient-reported outcome and cost measures will be completed by the participants at this moment, either on the phone with the research assistant or independently using a secured online survey link, depending on the participant’s preference. Text messaging and email reminders will be sent to improve participant retention [89]. We shall obtain information on health outcomes (eg, mortality) and physician charges via provincial databases. The charges billed by any physician 7 days after the initial visit will be used to estimate the costs of care for subsequent return visits and hospital admissions.

#### Outcome Measures

A value-based comparative assessment requires the simultaneous evaluation of health outcomes and costs. Our outcome measures were chosen from a guideline on the assessment of ED performance [90] and recent literature on patient experience assessment (Table 2) [91-95]. The initial visit, from arrival at a participating site to discharge, represents the unit of analysis for all outcome measures; however, the health system or patient costs incurred during the following week will be estimated and added to the cost of the initial visit. For participants assessed in person, the outcome will be scored per care setting and further stratified per discharge diagnosis and by province, using institution-specific costs for interprovincial cost comparisons. We will analyze the same outcome measures separately in the case of patients evaluated by telemedicine, as missing data (eg, vital signs) will prevent us from adjusting for the severity of their illness.

**Table 2:** Main study outcomes.

Outcome		Definition	Source
**Primary**			
	Incidence of return visit (O^a^)	Proportion of patients returning to any ED^b^ or outpatient clinic at 72 hours and 7 days after the initial visit [83,96-99]. An adjudication committee will review records of return visits to classify them as planned or unplanned and avoidable or unavoidable.	Follow-up call at 10 days
	Mean cost of care−the Ministry of Health perspective (C^c^)	The cost per care episode calculated by summing the costs of all care processes delivered to a patient during the initial visit plus the costs of return visits and admissions at 72 hours and 7 days.	Electronic medical records and provincial billing databases
**Secondary**			
	Median PROM-ED^d^ patients scores (O)	Developed and validated by team member SV, the PROM-ED questionnaire provides a measurement of patient-reported outcome expressed as scores for symptom relief, understanding of health concern, reassurance, and having a plan for care [91,94].	Follow-up call at 10 days
	Median scores on a PREM^e^ (O)	We adapted and are validating a tool from patient experience surveys used in EDs and primary care clinics in Ontario [100-102]. This tool evaluates the patient’s view of care delivery and measures various dimensions of patient experience relevant to all care alternatives, such as attitude of providers.	At the end of the initial visit
	Mean CoPaQ^f^ (C)	A questionnaire measuring patients’ and caregivers’ out-of-pocket expenses (eg, travel) and indirect costs (eg, loss of income) will be proposed to participants. This questionnaire was developed and validated by members of our team (ML, JG, SB) and further adapted for use in this study.	Follow-up call at 10 days
	Incidence of admission, intensive care unit, or mortality (O)	Proportion of patients who were admitted to the hospital or the intensive care unit or died because of one of the targeted respiratory conditions within 30 days [83,103] after the initial visit.	Provincial databases: Med-Echo, ICES, death registries
	Wait times	Median and mean length of stay and time spent waiting to see a physician.	Electronic medical records

^a^O: health outcome.

^b^ED: emergency department.

^c^C: health cost.

^d^PROM-ED: patient-reported outcome measure for ED.

^e^PREM: patient-reported experience measure.

^f^CoPaQ: cost-for-patient questionnaire.

To evaluate the quality of care in each group under study, compliance with practice guidelines (eg, corticosteroid prescription for asthma) for the treatment of respiratory diseases [104-109] will be compared (the full list of outcome measures is given in Multimedia Appendix 5 [83,91,94,96-103]). Return visits will be reported by the participants during the 10-day follow-up phone call. The Canadian Institutes of Health Research bridge grant obtained in April 2020 allowed our team to adapt questionnaires assessing patient perspective (Patient-Reported Experience Measure [PREM], patient-reported outcome measure for ED patients [PROM-ED], and cost-for-patient questionnaire [CoPaQ]) for use in any setting under evaluation. Their use for patients seen in person or by telemedicine in ambulatory patients with acute respiratory conditions will be validated further in the fall of 2020.

#### Sample Size

As our main analyses focus on patients assessed in person, our sample size calculation is based solely on their numbers. We estimate that the rate of return visit for ambulatory emergency conditions varies from 1% to 13% depending on the care setting [17,110,111]. To account for the potential similarity in outcomes among individuals in each of the 15 clusters, we assumed a realistic intracluster correlation of =0.02 based on previous studies and applied a correction to inflate our sample size calculation [112,113]. Using data from Campbell et al [28], at least 1485 patients (approximately 99 per cluster) will be needed to reveal a 5% difference in the proportion of return visits within 72 hours (eg, 5% vs 10%), assuming a 20% loss to follow-up and at least 30 participants per condition, based on multivariate logistic regression power analysis, type I error (α) at .05, and power at 80% (1−β). Assuming 240 recruitment days over a year at each site and the recruitment of at least 1 to 2 patients per day, our final cohort should include over 4000 patients and reach the minimal sample size in both participating provinces, which will allow for more robust comparisons and analyses.

#### Statistical Analysis

All main analyses will be conducted primarily on participants assessed in person in any of the care settings. Participants assessed by telemedicine will be analyzed and compared separately between sites where it is implemented. The value delivered at each participating site and on average in each care setting type will be illustrated with an operational effectiveness graphic [114]. Adjusted costs of care for acute ambulatory respiratory conditions will be plotted on the x-axis and adjusted return visits within 72 hours on the y-axis. Points closest to 0 on both axes represent the highest value of care (Multimedia Appendix 6). Indeed, the lower the return visit proportion and cost of care, the higher the value of care. Similar graphics will be used for patient-centered outcome measures. To compare EDs, walk-in clinics, and primary care physician practices, multilevel generalized linear models will be used with probability distributions adapted to the outcome under evaluation. To adjust for confounding (differences in case mix between care settings), an overlap weights approach [115,116] will be used, wherein each individual receives a weight factor that is proportional to the inverse of the probability of choosing a particular setting. Subjects that differ fundamentally between settings are attributed a weight of 0 and are thus excluded. This approach is of the greatest interest when groups are initially very different [116]. Intuitively, overlap weights create a pseudopopulation for which treatment is independent of measured confounders, thus mimicking a randomized trial of those confounders. Overlap weights have been shown to be more robust than conventional inverse probability weighting and matching based on propensity scores [115]. The weights will be estimated using a multinomial logistic regression model in which the dependent variable is the chosen care setting and independent variables are potential confounders or risk factors [117] for the outcomes identified in the literature: age [118,119], sex and gender [118,120], ethnicity [118,121-123], registration with a family physician [20,124], comorbidities (the Charlson index; number of regular medications) [118,125,126], asthma, FEV_1_ among patients with asthma, the Canadian deprivation index [127-129], patient perception of illness severity [130], and vital signs [131-135] as proxies for severity. The same independent variables will be used to adjust for differences in case mix between settings in the telemedicine cohort, excluding vital signs and FEV_1_. Multiple imputation will be considered as a possible means to adjust for these variables in this cohort. Overlap weights will be calculated using the values predicted by this model. We shall verify that the care setting groups are comparable according to the measured confounders after weighting by computing standardized mean differences. Differences below 10% will be considered to indicate good balance [117]. If residual imbalances are present, the weighting model will be revised. Once an appropriate balance is achieved, separate models for each outcome will be fitted to the weighted data, for which the care setting will be the only independent variable. The robustness of results with respect to unmeasured confounding will be assessed using the E-value [136,137]. Clustering by setting (eg, province, practice unit) will be taken into account using multilevel modeling (random intercept on province and practice unit). Reported cost estimates will be calculated with item-specific standardized costs (eg, Québec and Ontario average nurse unit cost). Patients referred to the ED from a participating outpatient facility but discharged home after ED assessment will be analyzed in the care setting group where they first presented, and the ED referral will be considered as a return visit. The costs of any return visits and admissions up to 7 days after the initial visit will be estimated separately and attributed to the care setting where the initial visit took place. Results of the 3 questionnaires from the patient perspective will be reported as proportions (PREM), mean costs (CoPaQ), and median scores (PROM-ED) and adjusted using the overlap weights approach. As patients seek care for symptoms, subanalysis based on presenting complaints instead of discharge diagnosis will be conducted to provide meaningful patient-oriented results. Other subanalyses will evaluate which patient profile (eg, gender [120], motivation for choosing a facility), and institutional characteristics (eg, access to x-ray) predict high quality and low costs, keeping in mind that our value assessment might not yield similar results for all subgroups or even within a group of patients with the same diagnosis. Statistical differences will be assessed with a significance threshold set at .05.

#### Sensitivity Analyses

To assess potential uncontrolled confounding of the results, sensitivity analyses will be conducted by excluding separately and concurrently the participants most likely to influence the effects of the 3 types of care settings: (1) ≥65 years; (2) with ≥1 comorbidity; (3) with either asthma or COPD; (4) with ≥1 regular medication; (5) with any abnormal vital signs; (6) in the lowest and highest quartile of the deprivation index; and (7) smokers. The analyses will be repeated using PMEWS instead of vital signs as a marker of illness severity. To control for a potential Hawthorne effect, the analyses will be repeated, with the first 3 months of recruitment excluded to focus on the data collected after the providers have become used to being observed.

## Results

### Study Preparation

From our pilot studies reported earlier until now, our team has made significant progress to reach its goal of identifying the care pathways providing the highest value to ambulatory emergency patients. We have assembled a very strong research team composed of patients, clinicians, administrators, and researchers. Together, we have created this paper. Two patient partners met with us regularly and provided helpful comments to make our research plan more patient centered. We have secured 14 of 15 planned participating sites. We have adapted the 3 patient-centered tools (PREM, PROM-ED, and CoPaQ) and are currently validating their use on ambulatory emergency patients whether they receive care in an ED, a walk-in clinic, or a primary care practice.

### Protocol Endorsement

Our protocol has been endorsed by the Network of Canadian Emergency Researchers (NCER). The broad support for our research initiative from leading Canadian organizations in emergency (NCER, Canadian Association of Emergency Physicians) and primary care (Réseau-1, Réseau de recherche axée sur les pratiques de première ligne, BeACCoN Ontario, Réseau sur les Innovations en soins de santé de première ligne et intégrés, Strategy for Patient-Oriented Research Unit), from the Ministries of Health of Ontario and Québec, and from organizations dedicated to improving health care throughout Canada (PULSAR, Canadian Institute for Health Information, Institut national d’excellence en santé et services sociaux, ICES) demonstrates the importance of the issue being addressed.

### Study Timeline

Phase 1 will begin in 2021 and will allow us to compare the cost of care from the public payer perspective in 3 different settings and 2 Canadian provinces. We expect that the results from this phase will be available in 2023. Phase 2 will begin in 2023 and will evaluate the value of the care in each setting under study. The final results will be published in 2025 and 2026. Our 4-year program covering the period of April 1, 2021, to March 31, 2025, is presented in a Gantt diagram available in Multimedia Appendix 7.

## Discussion

### Overview

Our unique multidimensional approach to examining the quality and cost of care using both patient and system perspectives will provide knowledge that will be helpful in determining whether EDs, walk-in clinics, or primary care practices offer the best value to patients with acute ambulatory respiratory conditions. We expect our study to yield tangible benefits for all stakeholders.

1. For guiding policy and decisions: Despite weak evidence, Canadian provinces have invested massively in alternative care pathways to get ambulatory patients with urgent care needs to rely less on hospital EDs. Data generated by the proposed study will have an immediate impact by providing hard evidence in support of health care planning decisions intended to improve the service quality/cost ratio and hence outcomes in the largest patient category.
2. For patients: Current policies are designed for statistically average ambulatory emergency patients without considering patient perspectives and the widely variable severity of each diagnosed illness. As the needs and preferences of patients with pharyngitis likely differ from those with exacerbated COPD, our stratified results per condition will enable policy makers to structure urgent care systems to provide better-adapted higher value services to each specific category of patients. Our comprehensive research initiative will bring patient preferences and perspectives into policy making.
3. For clinicians: Our study will be a powerful driver for quality improvement in all care settings involved. Care quality can vary considerably, and we hope to generate unique opportunities for valid and meaningful comparisons and for quality improvement initiatives throughout the country.

### Challenges and Mitigation Strategies

First, as patients choose their facility, those presenting at the 3 types of setting will likely represent different populations. However, we believe that the potential confounding bias due to self-selection of the care setting can be overcome using the overlap weights approach. Extensive testing of the robustness of our findings by sensitivity analyses should allow us to avoid reaching false conclusions under the influence of uncontrolled confounding. Second, the Québec and Ontario health systems might differ enough to yield results that will not be easy to generalize. When applicable, the sources of heterogeneity will be investigated. However, Canadian provincial health care systems have fundamental similarities that reduce the risk of poor generalizability. All are based on universal coverage; all suffer from a lack of integration between primary and urgent care resources [138,139]; institutions follow the recommendations of the same accreditation organizations; and care providers are trained according to the same standards and guidelines. Third, because of the pandemic, many outpatient clinics have ceased their activities or shifted to telemedicine. Our research plan already includes participants evaluated by telemedicine and will adapt easily to any increase in this practice. If the pandemic is still ongoing in November 2021 when phase 1 is launched, we will be able to collect financial data from participating institutions, which can be done remotely. Time measurement of care processes can be postponed until phase 2 in 2023, during which the recruitment of participants is planned. Finally, if the pandemic is still a factor in 2023, we will select clinics that continue to assess patients with acute respiratory disorders.

### Conclusions

Ambulatory emergency patients account for 30% to 60% of all ED visits in Canada. This burden on emergency care is now exacerbated by the COVID-19 pandemic. This category of patients is thought to be amenable to using walk-in clinics or primary care practices and is the focus of redirection strategies meant to decrease ED overuse. However, current knowledge is inadequate for reaching any firm conclusions about which care settings are best suited for this purpose. The aim of this study is to compare the value of the care that these patients receive in EDs, walk-in clinics, and primary care practices, thereby providing arm administrators and care providers with new and robust knowledge that will enable them to determine the best care setting for the management of respiratory ambulatory emergency conditions. We all agree that the system can only benefit from patients receiving timely care in the proper setting from the most suitable provider.

## Appendix

Multimedia Appendix 1 Participating sites per province and care setting.

Multimedia Appendix 2 Summary of overhead expenses.

Multimedia Appendix 3 Cost per time unit (Can $/min) of cost elements and estimated cost (Can $) of important care processes.

Multimedia Appendix 4 Steps of the time-driven activity-based costing method.

Multimedia Appendix 5 Complete list of study outcomes.

Multimedia Appendix 6 Example of an operational effectiveness graphic with hypothetical numbers.

Multimedia Appendix 7 Gantt diagram—value project.

## Abbreviations

BeACCoN: Better Access and Care for Complex Needs
CoPaQ: cost-for-patient questionnaire
COPD: chronic obstructive pulmonary disease
ED: emergency department
FEV1: forced expiratory volume in the first second
NCER: Network of Canadian Emergency Researchers
PMEWS: Pandemic Medical Early Warning Score
PREM: patient-reported experience measures
PROM-ED: patient-reported outcome measure for emergency department patients
URTI: upper respiratory tract infection
