# Supplementary material for: A Value-Based Comparison of the Management of Ambulatory Respiratory Diseases in Walk-in Clinics, Primary Care Practices, and Emergency Departments: Protocol for a Multicenter Prospective Cohort Study
Source: JMIR Res Protoc. 2021 Feb 22;10(2):e25619. doi: 10.2196/25619 (PMC7939947; doi:10.2196/25619)
Supplement: Multimedia Appendix 1 [file resprot_v10i2e25619_app1.pdf]

A1. Participating sites (expected n=15) per province and care setting

Province	Emergency department	Walk-in clinic	Primary care practice
**Québec**	▪ Centre Hospitalier de l'Université Laval (Quebec City)<br>▪ Centre hospitalier de Lanaudière (Joliette)<br>▪ Sacré-Coeur (Montreal)	▪ Clinique médicale Lévis-Métro (Quebec City)<br>▪ GMF Joliette (Joliette)<br>▪ GMF-R Concorde (Laval)	▪ Cité Médicale (Quebec City)<br>▪ GMF du Nord de Lanaudière (Joliette)<br>▪ GMF-R de la Cité de la Santé (Laval)
**Ontario**	▪ Ottawa Hospital (Ottawa)<br>▪ Kingston General Hospital (Kingston)	▪ St. Joseph Family Medicine Clinic (Ottawa)<br>▪ Medical Tree clinic (Kingston)	▪ Queen's Family Health Team (Kingston)
