# Supplementary material for: A Value-Based Comparison of the Management of Ambulatory Respiratory Diseases in Walk-in Clinics, Primary Care Practices, and Emergency Departments: Protocol for a Multicenter Prospective Cohort Study
Source: JMIR Res Protoc. 2021 Feb 22;10(2):e25619. doi: 10.2196/25619 (PMC7939947; doi:10.2196/25619)
Supplement: Multimedia Appendix 2 [file resprot_v10i2e25619_app2.pdf]

A2-a. Summary of overhead expenses at the participating emergency department[a,b], 2015-2016

Category	Can $ (hospital)	Allocation rule[c]	Can $ (ED)[d]
General administration	8,242,491	Proportion of the ED budget to the hospital budget	112,259
Financial administration	8,342,295		113,618
Staff administration service	3,923,829		53,441
Technical services administration	2,189,678		29,822
IT department	14,082,100		191,791
Human resources management	10,134,119		157,190
Supplies and services	4,256,013		83,000
Telecommunication	3,686,659		70,979
Transportation to home or long-term facility for patients 65 years and older	719,367	Attributed entirely to the ED	719,367
Archives	–	$8.91 per ED visit (75,929 visits in 2015-2016)	676,527
Food services	–	$5.67 per meal (44,642 meals)	252,100
Laundry	1,316,997	Proportion of ED laundry trolleys per day to the hospital laundry trolleys per day	263,399
Hygiene and sanitation−operational tasks	4,415,187	Proportion of ED surface area to the hospital surface area (m^2)	163,008
Hygiene and sanitation−functional tasks	331,106		12,224
Operation of the installations	4,730,723		103,864
Biomedical waste management	290,804	Proportion of ED biomedical waste mass to the hospital biomedical waste mass	11,632
Security	1,376,246	Proportion of ED volumetric space to the hospital volumetric space (m^3)	38,047
Maintenance (building, equipment and furniture)	6,832,644		166,836
Direction of social services	172,929	Attributed entirely to the ED	172,929
**Total**	**75,043,187**		**3,392,033**

[a]A currency exchange rate of US $1=Can $1.31 is applicable.
[b]Emergency department: ED; IT: information technology.
[c]Unless otherwise indicated, ED overhead cost = “hospital overhead cost” x “fraction allocated to the ED”.
[d]Overhead is allocated to the care of the whole population of ambulatory patients with acute conditions since all are treated in the same facility area by the same care providers. Using time-driven activity-based costing, overhead costs are then allocated to the care of each ambulatory patient based on the estimated time of contact between the patient and care resources.

A2-b. Summary of overhead expenses at the participating walk-in clinic[a,b], 2015-2016

Category[d]	Family medicine group and walk-in clinic (Can $)	Allocation rule	Walk-in clinic only (Can $)
General administration	9,000	Proportion of walk-in clinic patients to total patients visiting the clinic	4,320
Financial administration	5,400		2,592
Technical services administration	103,433		49,648
IT department	239,772		115,091
Human resources management	10,753		5,161
Supplies and services	80,585		38,681
Telecommunication	18,315		8,791
Archives	92,501		44,400
Laundry	2,622		1,259
Biomedical waste management	5,634		2,704
Operation of the installations	30,287		14,538
Maintenance (building, equipment and furniture)	79,247		38,039
Postage and messaging	19,696		9,454
**Total**	697,245		334,678

[a]A currency exchange rate of US $1=Can $1.31 is applicable.
[b]IT: information technology.
[c]Some categories were ignored as not applicable for the walk-in clinic.
