# Supplementary material for: A Value-Based Comparison of the Management of Ambulatory Respiratory Diseases in Walk-in Clinics, Primary Care Practices, and Emergency Departments: Protocol for a Multicenter Prospective Cohort Study
Source: JMIR Res Protoc. 2021 Feb 22;10(2):e25619. doi: 10.2196/25619 (PMC7939947; doi:10.2196/25619)
Supplement: Multimedia Appendix 3 [file resprot_v10i2e25619_app3.pdf]

**A3-a. Cost[a] per time unit (Can $/minute) of cost elements from our pilot study in the primary care practice (PCP) and in the emergency department (ED) in 2015-2016**

| Cost elements | PCP | ED |
|---|---|---|
| *Nurse* | 0.82 | 1.02 |
| *Auxiliary nurse/Beneficiary attendant* | 0.43 | 0.66 |
| *Clerks* | 0.29 | 0.56 |
| *Physicians* | 3.44 | 5.15 |
| *X-ray machine* | 0.65 | 0.21 |
| *Consumables* | 0.04 | 0.09 |
| *Overheads* | 0.32 | 0.22 |

[a]A currency exchange rate of US $1=Can $1.31 is applicable.

**A3-b. Estimated cost[a] (Can $) of important care processes from our pilot study in the primary care practice (PCP) and in the emergency department (ED) in 2015-2016**

| Care processes | PCP | ED |
|---|---|---|
| *Triage* | 8.70 | 9.50 |
| *Registration* | 1.10 | 2.25 |
| *Medical assessment* | 33.90 | 36.05 |
| *Chest X-ray*[b] | 21.25 | 21.20 |

[a]A currency exchange rate of US $1=Can $1.31 is applicable.
[b]Includes expenses related to technicians, interpretation by a radiologist, consumables, X-ray machine and overheads.
