# Supplementary material for: A Value-Based Comparison of the Management of Ambulatory Respiratory Diseases in Walk-in Clinics, Primary Care Practices, and Emergency Departments: Protocol for a Multicenter Prospective Cohort Study
Source: JMIR Res Protoc. 2021 Feb 22;10(2):e25619. doi: 10.2196/25619 (PMC7939947; doi:10.2196/25619)
Supplement: Multimedia Appendix 4 [file resprot_v10i2e25619_app4.pdf]

A4. Steps of the Time-driven activity-based costing method

1. Process mapping through discussion with local teams for each respiratory condition (eg, salbutamol in acute asthma) and resources involved (eg, respiratory therapist);
2. Validation of process maps and durations by on-field research assistants prospectively observing patients and measuring process duration using a time-motion software (UMT Plus (Laubrass))−**Figure 1**;
3. Estimate of cost per time unit of cost elements ($/min):
   i. Human resources (eg, nurses, physicians) or equipments (eg, X-ray machine)−**Figure 2**;
   ii. Consumable supplies (eg, gloves, needles, paper)–**Figure 3**;
   iii. Overheads (eg, building maintenance)–**Figure 4**;
4. Estimate of the cost of traceable supplies (eg, medication, laboratory testing);
5. Calculation of the average cost of each health care process–**Figure 5.**

Figure 1. UMT Plus (Laubrass)–configuration of time-motion software

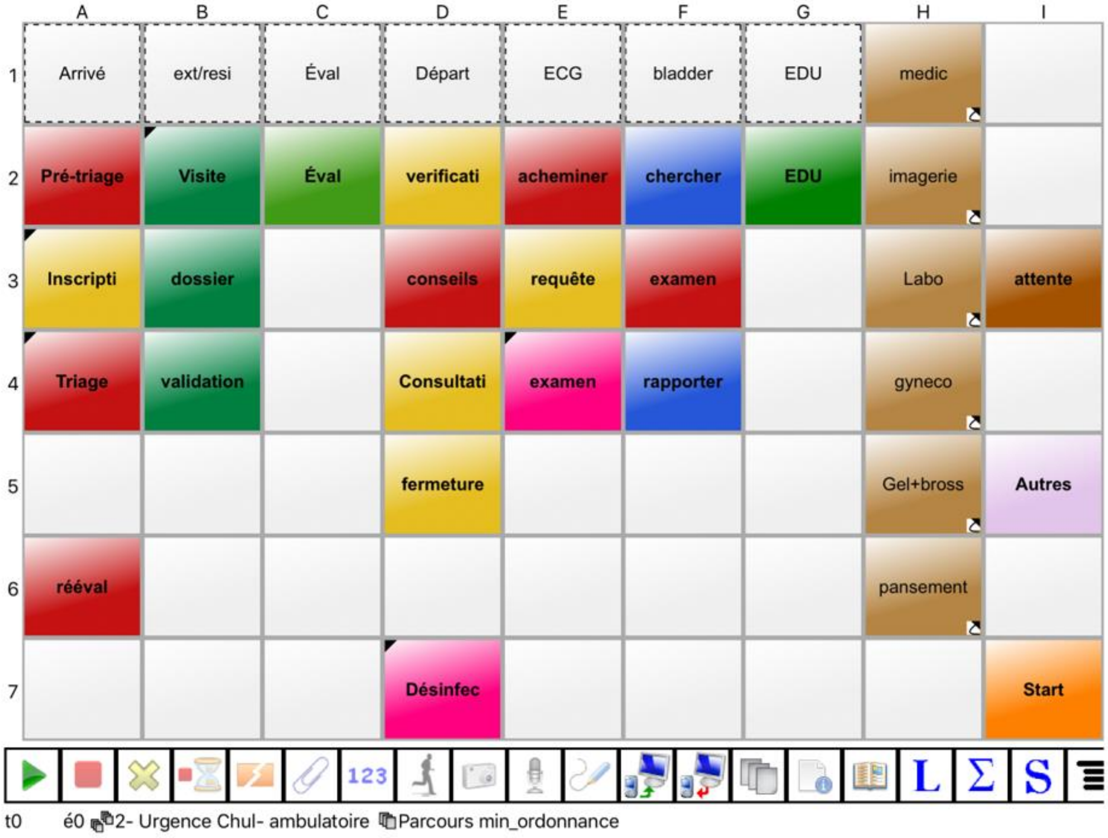

Figure 2. Unit cost (Can $) calculation for nurses in 2015-2016 at the CHUL ED[a-d]

$$\frac{\text{Total annual expenses for ED nurses}}{\text{Total annual minutes of availability to patient care (ED nurses)}}$$

$$\frac{\$8{,}414{,}750.92}{8{,}226{,}060\text{ min}} = 1.02\ \$/\text{min}$$

[a]Expenses include salaries, training stipends, benefits.
[b]Minutes exclude vacations, meetings and breaks.
[c]A currency exchange rate of US $1=Can $1.31 is applicable.
[d]CHUL: Centre Hospitalier de l'Université Laval; ED: emergency department.

Figure 3. Unit cost (Can $) calculation for consumable supplies used in 2015-2016 at the CHUL ED[a-d]

$$\frac{\text{Total annual cost of consumables}}{\text{Total annual minutes of availability to patient care (All ED professionals)}}$$

$$\frac{\$1{,}377{,}938.70}{15{,}162{,}187\text{ min}} = 0.09\ \$/\text{min}$$

[a]Consumables include all disposal supplies (e.g. needles).
[b]Minutes exclude vacations, meetings and breaks.
[c]A currency exchange rate of US $1=Can $1.31 is applicable.
[d]CHUL: Centre Hospitalier de l'Université Laval; ED: emergency department.

Figure 4. Unit cost (Can $) calculation for overheads in 2015-2016 at the CHUL ED[a-d]

$$\frac{\text{Annual overheads allocated to the ED}}{\text{Total annual minutes of availability to patient care (All ED professionals)}}$$

$$\frac{\$3{,}392{,}033.45}{15{,}162{,}187\text{ min}} = 0.22\ \$/\text{min}$$

[a]Overheads include institutional fixed and variable costs (e.g. human resources) imputed to the ED.
[b]Minutes exclude vacations, meetings and breaks.
[c]A currency exchange rate of US $1=Can $1.31 is
[d]CHUL: Centre Hospitalier de l'Université Laval; ED: emergency department.

Figure 5. Example of cost calculation for a care process (blood tests) using time-driven activity-based costing[a]

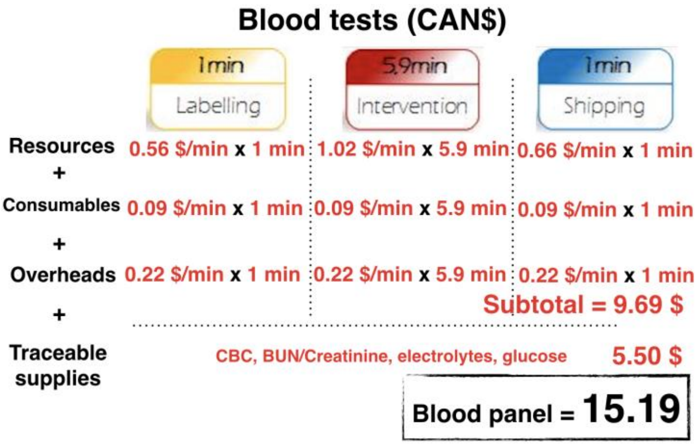

[a]A currency exchange rate of US $1=Can $1.31 is applicable.
