# Supplementary material for: A Value-Based Comparison of the Management of Ambulatory Respiratory Diseases in Walk-in Clinics, Primary Care Practices, and Emergency Departments: Protocol for a Multicenter Prospective Cohort Study
Source: JMIR Res Protoc. 2021 Feb 22;10(2):e25619. doi: 10.2196/25619 (PMC7939947; doi:10.2196/25619)
Supplement: Multimedia Appendix 5 [file resprot_v10i2e25619_app5.pdf]

A5. Complete list of study outcomes[a]

OUTCOME	DEFINITION	SOURCE
**PRIMARY**		
Incidence of return visit **(O)**	Proportion of patients returning to any ED or outpatient clinic at 72 hours and 7 days after the initial visit [87,100-103]. An adjudication committee will review records of return visits to classify them as planned/unplanned and avoidable/unavoidable.	Follow-up call at 10 days
Mean cost of care–Ministry of Health perspective **(C)**	Cost per care episode calculated by summing the costs of all care processes delivered to a patient during the initial visit plus the costs of return visits and/or admissions at 72 hours and 7 days.	Electronic medical records and provincial billing databases
**SECONDARY**		
Median PROM-ED scores **(O)**	Developed and validated by team member SV, the PROM-ED questionnaire provides a measurement of patient-reported outcome expressed as scores for symptom relief, understanding of health concern, reassurance and having a plan for care [95,98].	Follow-up call at 10 days
Median scores on a patient-reported experience measure (**O)**	We adapted and are validating a tool from patient experience surveys used in EDs and primary care clinics in Ontario [104-106]. This tool evaluates the patient’s view of care delivery and measures various dimensions of patient experience relevant to all care alternatives, such as attitude of providers.	At the end of the initial visit
Mean cost of disease–patient perspective **(C)**	A questionnaire measuring patients’ and caregivers’ out-of-pocket expenses (eg, travel) and indirect costs (eg, loss of income) will be proposed to participants. This questionnaire was developed and validated by members of our team (ML, JRG, SB) and further adapted for use in this study.	Follow-up call at 10 days
Incidences of admission, intensive care unit or mortality **(O)**	Proportions of patients who were admitted to hospital or to the intensive care unit, or died of one of the targeted respiratory conditions within 30 days [87,107] after the initial visit.	Provincial databases: Med-Echo, ICES, death registries
Wait times	Median and mean length of stay and time to see a physician.	Electronic medical records
Incidence of oral corticosteroid prescription	Proportion of patients with exacerbated asthma or COPD who received a prescription for oral corticosteroids.	Electronic medical records
Incidence of antibiotic prescription	Proportions of patients with URTI, otitis media, flu or bronchitis who received a prescription for antibiotics.	Electronic medical records
Incidence of chest X-ray use	Proportions of patients with URTI, bronchitis and asthma who had a chest X-ray performed.	Electronic medical records
Compliance to guidelines on use of antibiotics	Proportions of compliance to provincial recommendations of antibiotic prescriptions for pneumonia, tonsillitis and acute exacerbation of COPD.	Electronic medical records
Incidence of diagnostic spirometry prescription	Proportions of spirometry prescribed for long-term >40-year-old smokers (current or past) undiagnosed with COPD who present for an acute lower respiratory tract infection.	Electronic medical records

[a]O: health outcomes; ED: emergency department; C: health costs; COPD: chronic obstructive; pulmonary disease; URTI: upper respiratory tract infection.
