# Supplementary material for: A Value-Based Comparison of the Management of Ambulatory Respiratory Diseases in Walk-in Clinics, Primary Care Practices, and Emergency Departments: Protocol for a Multicenter Prospective Cohort Study
Source: JMIR Res Protoc. 2021 Feb 22;10(2):e25619. doi: 10.2196/25619 (PMC7939947; doi:10.2196/25619)
Supplement: Multimedia Appendix 6 [file resprot_v10i2e25619_app6.pdf]

A6. Example of an operational effectiveness graphic with hypothetical numbers[a]

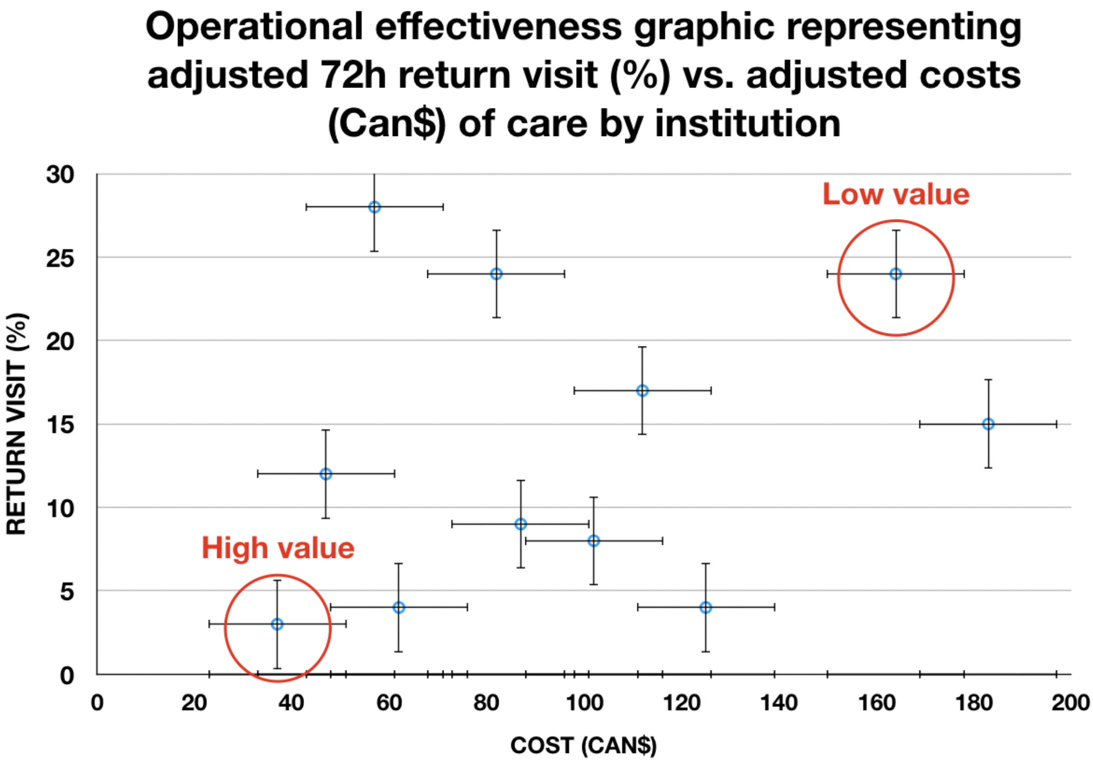

[a]A currency exchange rate of US $1=Can $1.31 is applicable.
