# Supplementary figures and images for: A Value-Based Comparison of the Management of Ambulatory Respiratory Diseases in Walk-in Clinics, Primary Care Practices, and Emergency Departments: Protocol for a Multicenter Prospective Cohort Study
Source: JMIR Res Protoc. 2021 Feb 22;10(2):e25619. doi: 10.2196/25619 (PMC7939947; doi:10.2196/25619)

**A7. Gantt diagram: 4-year timeline–Value Project**

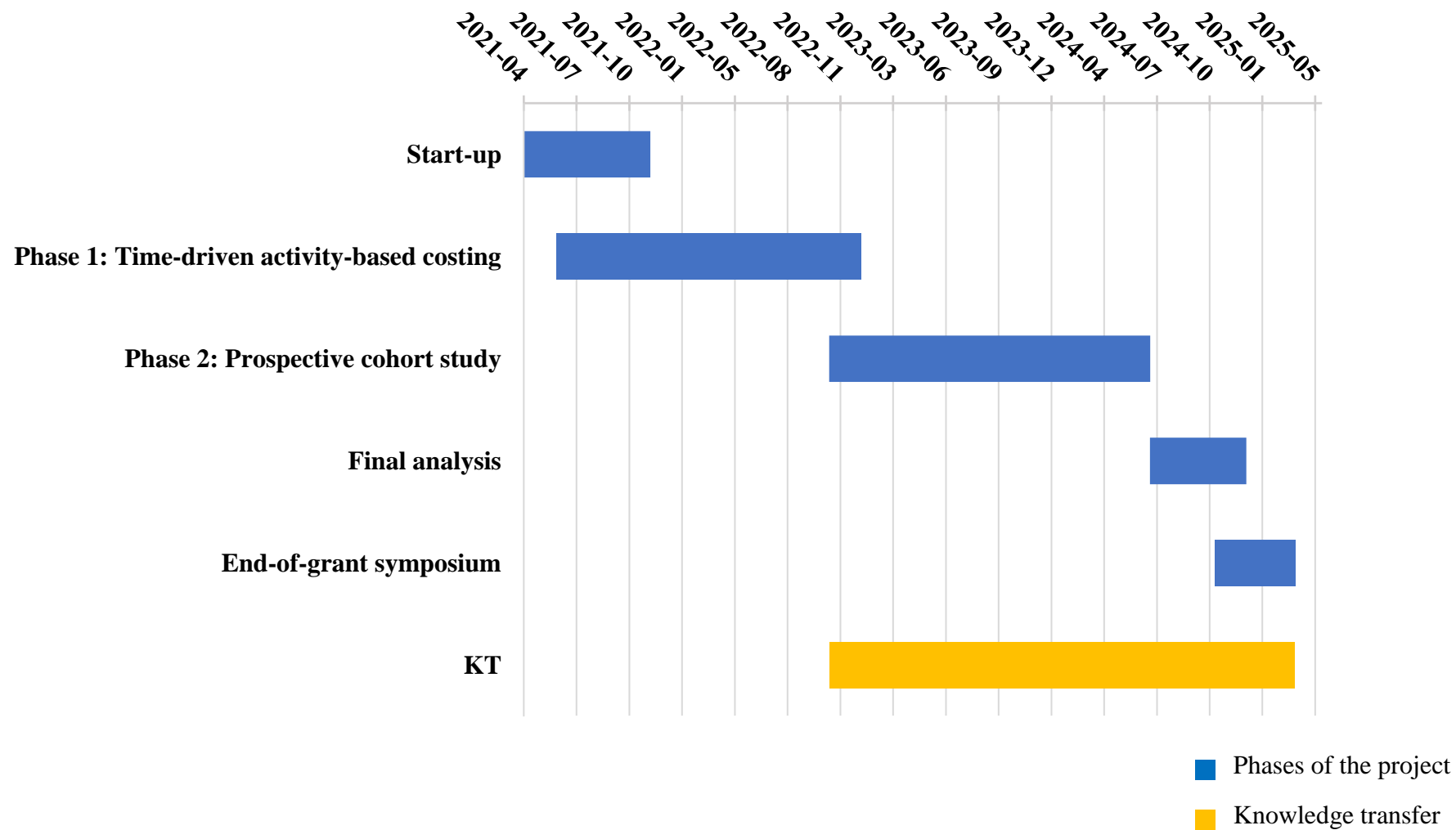

Supplement: Multimedia Appendix 7 [file resprot_v10i2e25619_app7.pdf]
